# Knowledge of dental faculty in gulf cooperation council states of multiple-choice questions’ item writing flaws

**DOI:** 10.1080/10872981.2020.1812224

**Published:** 2020-08-24

**Authors:** Mawlood Kowash, Hazza Alhobeira, Iyad Hussein, Manal Al Halabi, Saif Khan

**Affiliations:** a Pediatric Dentistry Department, Hamdan Bin Mohammed College of Dental Medicine, Mohammed Bin Rashid University of Medicine and Health Sciences, Hail, United Arab Emirates; b Restorative Dentistry Department, Hail University, Hail, Saudi Arabia; c Basic Dental and Medical Sciences Department, Hail University, Hail, Saudi Arabia

**Keywords:** Dental education, assessment, multiple-choice questions, dental faculty, item-writing flaws (IWFs)

## Abstract

Multiple-Choice Questions provide an objective cost/time effective assessment. Deviation from appropriate question writing structural guidelines will most probably result in commonly ignored multiple-choice questions writing flaws, influencing the ability of the assessment to measure students’ cognitive levels thereby seriously affecting students’ academic performance outcome measures. To gauge the knowledge of multiple-choice question items writing flaws in dental faculty working at colleges in Gulf Cooperation Council (GCC) countries. A cross-sectional short online *Survey Monkey^TM^* multiple-choice questions-based questionnaire was disseminated to dental faculty working in GCC countries during the academic year 2018/2019. The questionnaire included five test incorrect (flawed) multiple-choice questions and one correct control question. The participants were asked to identify flawed multiple-choice question items from the known 14 items writing flaws. Out of a total of 460 faculty, 216 respondents completed the questionnaires, 132 (61.1%) were from Saudi Arabia, while numbers of participants from United Arab Emirates, Kuwait and Oman were 59 (27.3), 14 (6.5%) and 11 (5.1%) respectively. Majority of participants were male (n = 141, 65.9%) compared to 73 females (34.1%). Eighty percent of the participants possessed more than five years of teaching experience. Assistant professors constituted the majority (43.3%) of the academic positions participating in this study. The overall fail rate ranged from 76.3% to 98.1% and almost 2/3^rds^ of the participants were unable to identify one or more of the flawed item(s). No significant association was observed between the demographics (age, region, academic position and specialty) and knowledge except that of participant’s gender (p < 0.009). GCC dental faculty demonstrated below average knowledge of multiple-choice question items writing flaws. Training and workshops are needed to ensure substantial exposure to proper multiple-choice question items construction standards.

## Introduction

*Multiple-choice questions* (MCQs) are an effective and easy method of assessment when deployed on their own or in conjunction with other types of educational assessments due to several advantages such as reliability, content validity, reduced reliance on students’ skills of writing and self-expression [1]. Effective and well-constructed MCQs are also considered a time- and cost-effective assessment tool that can test many candidates, cover extensive content topics, and have better objective grading accuracy and consistency compared to other forms of assessment [2]. In addition to the fact that high quality effective MCQs may be employed to measure knowledge and understanding, MCQs may be designed to measure the analytical ability and practical applicability of concepts [1]. However, to be effective, MCQs should be of high quality and free of *item writing flaws* (IWFs). Regrettably, several studies reported that most examiners and instructors fail to follow generally accepted guidelines of constructing effective MCQs [3–5]. *Lower* cognitive *levels* MCQs such as recall of facts tend to test the lower cognitive levels of Bloom’s taxonomy and compromise the reliability and validity of the question items as claimed by some previous studies [6–8]. A recent study concluded that question items with high-level taxonomy performed better in discrimination indices [9]. The relationship between cognitive level and MCQ has been studied before and the results show that case-based vignettes can achieve higher cognitive levels with better psychometric properties [10].

Scouller investigated the effect of evaluation methods on students’ learning techniques and showed that generally, examinees were more likely to adopt a superficial style when the evaluation doctrine was based solely on recollection of facts [11]. The same study found that students and trainees were more likely to implement a deeper approach for learning if the test questions required analytical higher levels of cognitive abilities [11]. Several studies reported that MCQ-IWFs are frequently observed in health education assessment examinations [4,12,13]. For example, Downing reported that 36–65% of the items were flawed in four basic science tests [4].

Recently, Melser *et al*. concluded that introducing a faculty training program for writing high-ordered MCQ improved cognitive levels of MCQ items constructed by the faculty [14].

The inclusion of ‘except’ or ‘not’ in the lead-in, tricky or unfocussed stems, use of absolute terms (never, always, none), ‘all and none of the above’ as an answer choice option and giving opportunities for students to use convergence strategies, which allow them to answer the question by recognising that the correct answer includes common elements of other options. The aforementioned constituted the most common MCQ-IWFs [4,8,15].

To our knowledge there are no international studies generally, and none in the Gulf Cooperation Council States (GCC) states specifically; [Kingdom of Saudi Arabia (KSA), United Arab Emirates (UAE), Qatar, Kuwait, Oman and Bahrain], that have investigated dental faculty’s knowledge of the generally accepted principles and guidelines of constructing effective MCQ items. Therefore, it seemed appropriate to evaluate GCC dental school faculty’s ability to identify common MCQ-IWFs and suggest ways to improve their MCQ item writing skills.

## Methods

### Design of study and target population

A cross-section study design using a *Survey Monkey^TM^* question format, was conducted in the only GCC countries that have undergraduate dental schools namely: KSA, UAE, Kuwait, and Oman. The total number of undergraduate dental schools in the GCC are 30 public and private schools. The sample size was calculated according to the average number of 16 full-time faculty in each dental school constructing MCQ items, therefore, the approximate number of faculty would be around 480. Using the formula of Cochran’s sample size calculation for cross-sectional design (at 95% confidence interval and a margin of error of 5%), the calculation yielded a sample size of 218 however, 216 participants’ questionnaires were complete and statistically analyzed. The study’s ethics were reviewed by the Institutional Review Board (IRB) of Mohammed Bin Rashid University for Medicine and Health Sciences (MBRU), was granted an IRB exemption (# MBRU-IRB-2018-010).

### Questionnaire

The structured questionnaire was divided into two parts. The first part included the demographic data of the participating faculty and the second part comprised of six questions, five of which included anyone (or more) type of IWF(s) based on accepted guidelines [5,16]. The sixth MCQ was the effectively and adequately designed (control) question (Table 1). The participating faculty were prompted to identify if the MCQ was flawed or not. If the participant identified an MCQ as flawed, then he/she was prompted to classify the flaw from a 14-item drop-down menu (Table 2) of standard MCQ-IWFs [5].

**Table 1.** The five test (flawed) and one control (effective) MCQs.

Question	Stem	Lead in	Options
1(Test)	An 8 -year-old healthy boy presented with uncomplicated crown fracture and extrusion injury of his maxillary left incisor (tooth 21) following a fight at school. You have been asked to provide a medicolegal report stating the percentage of pulp survival of the injured tooth.	What is the most likely percentage of pulp survival of tooth 21 according to the available literature?	A. Less than 40% B. 50 to 60% C. Greater than 50% D. 90% E. 75%
2 (Test)	All the following are behavior management techniques in pediatric dentistry EXCEPT:	_	A. Tell, Show, Do B. Distraction C. Reinforcement D. Modelling E. Pulp vitality testing
3 (Test)	A 5-year-old healthy and reasonably cooperative child with multiple asymptomatic decayed teeth in all quadrants.	Which region you should chose that is most easily anaesthetized with the least discomfort?	A. Maxillary molar region B. Maxillary incisor region C. Mandibular molar region D. Mandibular incisor region E. None of the above
4 (Test)	In regard to intravascular injections and the effects of local analgesia	Which of the following statements is correct?	A. Intra-venous injections can produce systemic bradycardia B. Intra-arterial injections are more common than intravenous injections C. The consequences of an intra- arterial injection can be alarming. Such effects range from local pain and cutaneous blanching D. The consequences of an intra- venous injection can be alarming. Such effects range from local pain and cutaneous blanching
5 (Test)	Patients with dentinogenesis imperfecta:	_	A. Have bulbous crowns and obliterated root canals B. Always on bisphosphonate treatment C. Their condition is not hereditary D. Never suffer from osteogenesis imperfecta
6 (Control)	A 16- year old boy suffered a deep stab wound which caused severe bleeding. It involved the angle of the mouth and continued upward along the side of the nose and ended at the medial canthus (inner corner) of the left eye.	What artery is most likely to be involved?	A. Common carotid artery B. Internal carotid artery C. External carotid artery D. Facial artery E. Maxillary artery

**Table 2.** Summary of technical item flaws.

***Flaws Related to Testwiseness*** **Grammatical cues –** one or more distractors do not follow grammatically from the stem **Logical cues –** a subset of the options is collectively exhaustive **Absolute terms –** terms such as ‘always’ or ‘never’ are in some options **Long correct answer –** correct answer is longer, more specific, or more complete than other options **Word repeats –** a word or phrase is included in the stem and in the correct answer **Convergence strategy –** the correct answer includes the most elements in common with the other options.
***Flaws Related to Irrelevant Difficulty*** 7. Options are long, complicated, or double 8. Numeric data are not stated consistently 9. Terms in the options are vague (e.g., ‘rarely,’ ‘usually’) 10. Language in the options is not parallel 11. Options are in a nonlogical order 12 ‘None of the above’ is used as an option 13. Stems are tricky or unfocussed 14. Except or Not in the lead-in

The questionnaire was reviewed and approved by a medical education expert at MBRU, Professor Dave Davis and was based on previous published research [15]. The questions were piloted among 10 faculty attending the Ottawa education conference in Abu Dhabi Emirate on the 13^th^ of March 2018.

### Statistical analysis

SPSS for windows version 24.0 (SPSS Inc., Chicago, IL) was used to perform statistical analysis. Descriptive statistics were used to describe categorical and continuous variables. Cross-tabulation between demographics data of with participant responses to the MCQs was conducted using χ2-square and Exact Fischer’s test when appropriate (P-value < 0.05 was considered as significant).

## Results

Two hundred and sixteen dental teaching faculty in the GCC completed the survey out of the total number of 480 participants yielding a response rate of 45%. A very high proportion (n = 132, 61.1%) hailed from KSA and were males, 59 (27.3%) from the UAE, 14 (6.5%) from Kuwait and 11 (5.1%) from Oman. Two-thirds of the participants were male and most of them were between 30 and 50 years of age. A majority (80%) had more than five years teaching experience. Assistant professors constituted most of the participants. The participants’ demographics are summarized in Table 3.

**Table 3.** Participant demographics.

Age Distribution (%)	20–29	30–39	40–49		50–59		60–69	
	2.3	35.3	38.1		16.3		7.4	
Teaching Experience (%)	0–5	6–10	11–15		16–20		>20	
	2.3	35.3	38.1		16.3		0.5	
Academic Position (%)	Lecturer	Assistant Professors	Associate Professors		Professors		Others	
	16	44	19		15		6	
Specialty distribution (%)	Basic Dental Sciences	Endodontics	Oral Surgery	Restorative Dentistry	Orthodontics	prosthodontics	Pediatric Dentistry	Periodontics
	13	5	9	12.1	7	10.7	17	5.6

**Figure 1.**
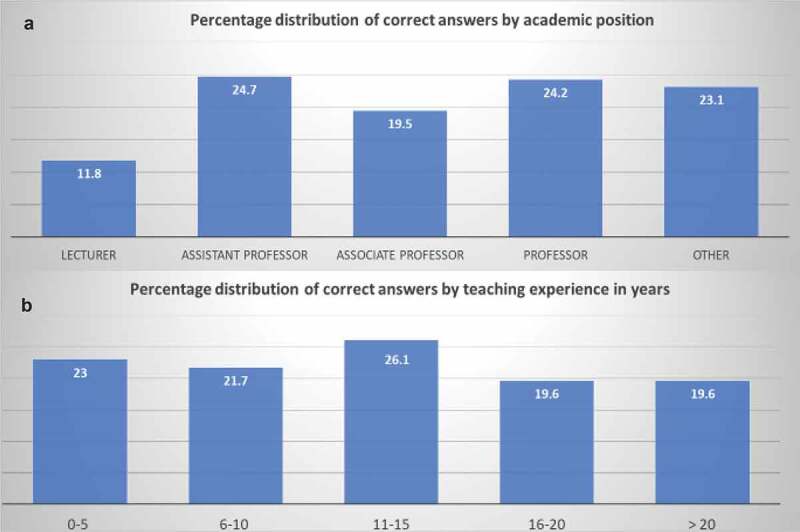
Percentage distribution of correct answers **A**: by academic position, **B**: by teaching experience.

Nearly half (48%) of participants incorrectly answered all the questions. Overall, 17.3% could answer only one question correctly, while 15.4%, 12.1% and 6.5% participants were able to correctly answer two, three and four questions, respectively. Only 0.5% participants answered all the questions correctly. Distribution of correct answers by teaching experience and academic position is summarized in Figure 1.

Ability of the participants to discover and consequently identify the type of MCQ-IWFs was very low; 78.1% of the participants could not discover that Question No #1 was flawed (MCQ-IWF type: *numerical data not stated consistently)*; 76.3% participants considered Question No #2 either correct or were not able to characterize the type of flaw, (MCQ-IWF type: *except in the lead and implausible options*); almost all of the participants (98.1%) were not able to characterize question #3 as flawed or could not classify it into the correct IWF (MCQ-IWF type: *use of none of the above)*; 73% of the participants failed to identify/characterize the IWF in question No #4 (MCQ-IWF type: *convergence strategy*); and finally 96.3% of the participating dental faculty could not recognize or characterize the flaw in question #5: (MCQ-IWFs type: *use of absolute term-never” and ‘ short unfocused stem’)*. In addition, it was noted that 64.7% of the participants wrongly considered the correct control question #6 as flawed (Table 4).

**Table 4.** Type of IWFs and distribution of wrong answers in each question.

Question Nr	Type of Flaw	Wrong answer (%)
1	*numerical data not stated consistently*	78.1
2	*no lead in, implausible options and use of except in the lead*	76.3
3	*use of none of the above*	98.1
4	*convergence strategy, long option*	73
5	*‘use of absolute term-never’, ‘short unfocused stem’ and no lead in*	96.3
6 (control)	None	64.7

**Figure 2.**
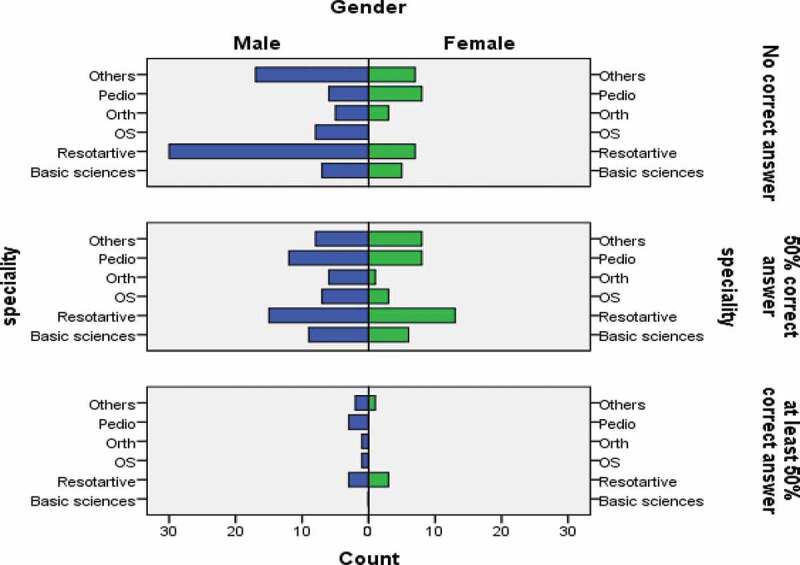
Distribution of correct/incorrect answers of female and male faculty by specialty. (OS: Oral Surgery; Basic Sciences: Basic dental sciences; Orth: Orthodontics; Pedio: Pediatric dentistry; Restorative: Restorative dentistry.).

Demographics of participants and their responses were found to have no significant association except for gender and response to question No #1. Females’ responses were more accurate when compared to males (p = 0.009). Distribution of correct/incorrect answers of female and male faculty by specialty is summarized in Figure 2.

Finally, in response to the question ‘do you have any formal MCQ items writing training?’, 61 (28%) did not have, 75 (35%) had and 79 (37%) did not answer.

Using logistic regression analysis, the difference between those who had and had not attended a previous training course in MCQ writing (as dependent variable) was statistically significant (p = 0.048) when it was adjusted to other variables (teaching experience and academic position). The independent variables were: experience (less than 10 years and more than 10 years), current academic rank (lecturer and assistant professor vs associate professor and full professor) and the knowledge variables coded as (no knowledge, some knowledge, and reasonable knowledge). Finally, approximately two-thirds of the participants (n = 136, 63%) expressed interest in attending MCQ items writing courses/workshops.

## Discussion

The present study attempted to determine the knowledge of dental faculty across GCC regarding the identification of flawed MCQs. The study participants were well distributed across the GCC countries. The regionwide participation was proportional to the area of the countries and the number of dental schools in these GCC countries. No bias was observed in ‘age’ and ‘teaching experience’ of the participating dental faculty since both age and teaching experience followed a normal bell-shaped distribution pattern (Table 3). The study demonstrated the highest contribution by assistant professors (44%, Table 3). This reflected the unbiased contribution since the participating colleges possessed the highest proportion of assistant professors when compared to lecturers, associate and full professors individually. A higher participation of male dental faculty (65%) in the study possibly reflects the higher proportional ratio of males/females in the GCC dental colleges [17]. Specialty wise, participation was unbiased since the percentage contribution of each dental specialty appeared to be proportional to the corresponding staff ratio of the participating colleges (Table 3). It can be reasonably concluded that the present study was unbiased and evenly distributed in all attributes (age, sex, region, academic position, and specialty) of dental faculty participants.

Majority of participants (~48%) were not able to recognize the flawed MCQs (refer to results section for detailed percentage distribution). This represents the poor ability of the participants to recognize MCQ-IWFs and thereby possible inability to construct proper MCQs as per the prescribed standards [18]. Figure 1 highlights that there was no significant difference in distribution of correct answers on the basis academic position when compared to teaching experience.

Several studies have reported the percentage of flawed MCQs in medical/other health sciences examinations and their effects on student’s learning [4,6,8]. However, none of these studies had directly attempted to determine the knowledge of the teaching faculty (responsible for designing MCQs at the institutional level) regarding standard MCQ construction and associated MCQ-IWFs. Our study tried to fill this gap by measuring the knowledge of the dental faculty.

Application of distractors such as ‘none of the above’ and use of absolute negative terms together with short unfocussed question stem represented the most frequent MCQ-IWFs ignored by the dental faculty (Table 4). This observation coincides with the findings in other studies [6,13,19]. Wherein the said MCQ-IWFs (use of the none of the above and weak question stem) constituted the major percentage of the flawed MCQs. A weak question stem may be a consequence of English being the second language for the MCQ author. The use of simplicity and clarity in the language used in both the stem and the options is highly recommended in writing effective high cognitive level MCQs as it leads to the reduction of the influence of the level of reading abilities on student performance [20–22].

The use of ‘none of the above’ as a distractor is not recommended since it only qualifies the students’ incorrect option identification ability. In case ‘none of the above’ is the right answer, the MCQ writer must ascertain that no exceptions exist for the rest of the distractors [18–22].

The current study did not include all possible MCQ-IWFs in the mock questionnaire. However, this probably had no or minimal effect on the study objective since the participants had chosen from a generally accepted 14-item IWFs list [5,16] and the sample of the five flawed MCQs used the most commonly reported IWFs namely: the use of ‘except’ or ‘not’ in the lead-in, the use of absolute terms like ‘never’ in the options and tricky or unfocussed stems and giving opportunities for students to use convergence strategies [4,8,15].

Another observation in the present study was that the statistically significant difference in the recognition of flawed MCQs and associated IWFs by female dental faculty. Female participants in our study appeared to have a better understanding of MCQ construction standards and IWFs when compared to males. The reason why female faculty performed better than males was not investigated, however, it could be an incidental finding. It has been reported by Schmitt *et al*. [23] that in terms of the Big Five taxonomy, females consistently report higher levels of openness to experience, agreeableness (*friendly/compassionate* vs. *challenging/callous*) and neuroticism (*sensitive/nervous* vs. *resilient/confident)*. Females have also been found to report higher levels of conscientiousness (*efficient/organized* vs. *extravagant/careless)* [23].

Gender-based classification of specialty for the knowledge of MCQs IWF is Figure 2. Male and female participants of the restorative specialty appeared to possess relatively better understanding of IWFs. However, no significant difference is observed between the participants.

There is an urgent need to improve GCC dental faculty MCQ items’ writing skills, which can be achieved through short or long MCQs’ writing workshops. A one full week study for medical faculty reported an increase in faculty confidence and knowledge in writing effective high level MCQs items [24]. The week-long workshop raised feasibility concerns due to its practical implications. AlFaris *et al*. in 2015 assessed the effectiveness and the impact of a one-day MCQ-writing workshop on the quality of MCQ writing in a dental school. The results demonstrated significant improvement in the knowledge of the participating faculty and the quality of MCQ writing. Furthermore, all the participating faculty were highly satisfied with the training [25]. Another study conducted by Dellinges and Curtis in 2017 found that a short duration training session significantly improved the quality of in-house MCQ item-writing of US dental school faculty [26].

Individual and institutional barriers often lead to limited outcomes of short courses and workshops of MCQ items writing [27]. In contrast, longitudinal faculty development workshops were reported to be successful in improving the quality of the MCQs items writing skills of the faculty that leads to students’ high proficiency levels [28].

A recent study emphasized the importance of a more personalized approach to enhance the skills on MCQ writing by giving timely feedback which is a good strategy for developing the faculty’s ability on writing good quality items [29].

Limitations of study included general problems attributed to questionnaire surveys such as a biased and overly simple view of reality, the problems related to validity and reliability of results which may result in increasing the risk of both representation and measurement errors. The primary limitation of this study is the small number of questions (only six) exposed to the participants. This was done to entice the participants to complete a small questionnaire, since long questionnaires may result in the loss of interest of the participating faculty members.

## Conclusions

The results of the study demonstrated poor knowledge of GCC dental faculty in identifying MCQ-IFWs. Almost half of the participants were unable to identify any IWFs in the MCQs. Female participants were significantly better in IFW identification compared to their male counterparts. Faculty who attended MCQ item writing courses performed better and hence faculty development in the area of writing structured accurate MCQs in the GCC countries is recommended.

## Data Availability

We are happy to provide the raw data that this study was based on upon request from the corresponding author. The data are not available in the public domain because of confidentiality and ethical reasons.
